# A large staghorn stone diagnosed and managed in an asymptomatic patient using the “Kidney Injury Test (Kit)” spot urine assay: A case report

**DOI:** 10.1016/j.eucr.2021.101854

**Published:** 2021-09-21

**Authors:** Leslie Bernal Charondo, Fadl Hamouche, Reuben D. Sarwal, Minnie M. Sarwal, Thomas Chi, Marshall L. Stoller

**Affiliations:** a School of Medicine, University of California, San Francisco, USA; b Department of Urology, University of California, San Francisco, USA; c Nephrosant, Brisbane, USA

**Keywords:** Nephrolithiasis, Kidney injury test (KIT), Urinary biomarker, Staghorn, Cell-free DNA

## Abstract

The Kidney Injury Test (KIT) Stone-Score provides an objective measure of stone burden. Unlike urinary supersaturation the KIT Stone-Scores assess underlying stone disease rather than urinary solute composition. We report a case of a 43-year-old woman with no history of nephrolithiasis who underwent an elective, voluntary KIT assay and was diagnosed with a large staghorn renal stone after an unanticipated markedly elevated score. This clinical scenario highlights the potential future use of the non-invasive urinary KIT assay as a reliable non‐invasive tool to detect and monitor urinary stone disease.

## Highlights

• KIT assay consists of a panel of six urinary biomarkers measured on a random urine sample.
• Healthy asymptomatic patient was diagnosed with an unanticipated staghorn kidney stone after an elevated elective KIT assay.
• For healthy patients, the threshold for the KIT Stone-Score was determined to be less than or equal to 30.
• Urinary KIT normalized 6-months post-operatively.
• KIT assay predicts the future risk of recurrence and risk stratify kidney stone patients.

## 1 Introduction

Nephrolithiasis is a common problem worldwide. Radiation exposure may become significant when taking into consideration the imaging required in the diagnosis and follow-up of kidney stones.^1^ The development of a spot urine test was developed to detect stone recurrence and correlate with stone burden.

The Kidney Injury Test (KIT) assay consists of a panel of six urinary biomarkers and has been validated for early detection of kidney injury and nephrolithiasis.^2^ Furthermore, the scaled KIT Stone-Score readily discriminated individuals with current or prior radiographically confirmed kidney stones from healthy non-stone formers. A healthy asymptomatic patient was diagnosed with an unanticipated large staghorn kidney stone after an elective KIT assay revealed a markedly elevated score.

## 2 Case presentation

**Fig. 1:**
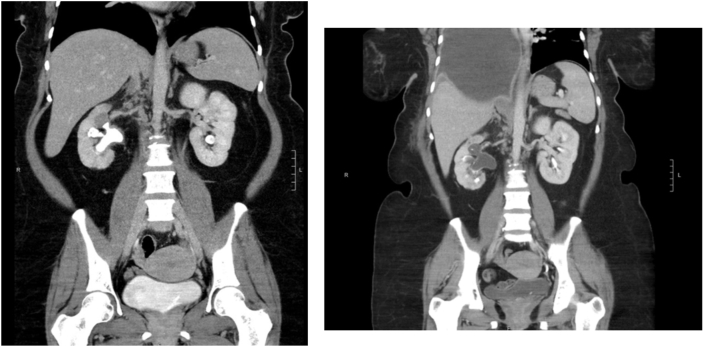
A large right staghorn calculus and moderate hydronephrosis at upper pole (A). Final CT scan with contrast showing minimal stone residuals on day of discharge (B).

A 43-year-old female with no previous personal or familial history of kidney stones underwent an elective KIT urinary test as part of a non-stone former control group for a separate unrelated study. Surprisingly, her urine KIT assay revealed six abnormally elevated proteins with a combined KIT Stone-Score of 75 (Table 1). She had no hypertension but was noted to have obesity (BMI 36), iron deficiency anemia, and vitamin D deficiency. Her only medication was an oral contraceptive, and her clinical exam was unremarkable. Further work-up consisted of a computerized tomography (CT) scan to assess stone burden and it revealed a complete right staghorn calculus with moderate right-sided hydronephrosis (Fig. 1A). As part of the pre-operative work-up, the urinalysis identified frank leukocyturia and microscopic hematuria. A subsequent urine culture identified an E. coli infection that was appropriately treated with oral antibiotics. The patient underwent a right percutaneous nephrolithotomy (PCNL) with clearance of all visible stones.

**Table 1:** KIT stone assay biomarker values.

	Healthy (Non-Stone Former)	Healthy (Non-Stone Former)	Stone Former	Patient: Pre-Op	Patient: Post-Op
cfDNA (ng/mL)	3.79	1.67	12.13	465.37	0.86
mcfDNA (ng/mL)	3.27	2.11	19.92	90.2	12.8
Clusterin (ng/mL)	188.22	34.0	52.79	101.96	107.68
CXCL10 (pg/mL)	4.28	0.51	1.09	4.67	0.54
Total Protein (ug/mL)	56	27	59	149	0.1
Creatinine (mg/dL)	42.2	34	67.8	29	42.8
KIT Stone-Score	3.5	7	70	75	9.4

**Fig. 2:**
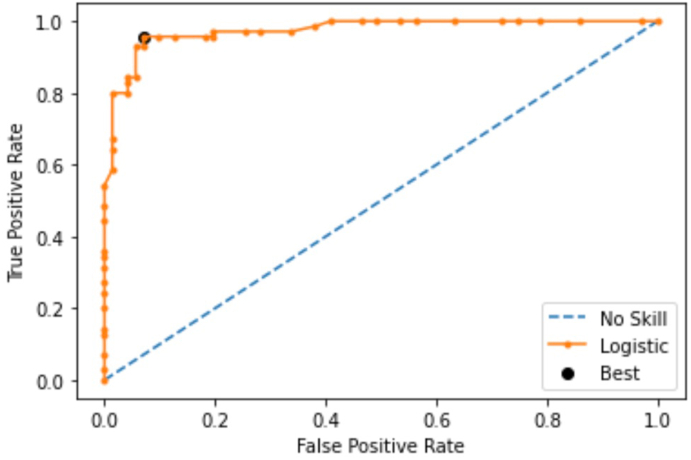
ROC analysis in healthy patients determined the KIT Stone-Score threshold to be less than or equal to 30, with an AUC of 0.94.

A follow-up CT scan identified a residual stone fragment (10 mm) in the right lower pole with resolution of the previous hydronephrosis (Fig. 1B). A second stage right ureteroscopy with laser lithotripsy (URS-LL) rendered her stone free. Her KIT results normalized 7 months post-operatively with similar results to non-stone formers (Table 1). For healthy patients, the threshold was estimated using a receiver operator characteristic curve (ROC) analysis for the KIT Stone-Score was determined to be less than or equal to 30, with an area under the curve (AUC) of 0.94 (Fig. 2).

## 3 Discussion

Repeat patient exposure to radiation from scans and their associated costs are particularly problematic in patients with recurrent stone disease. Patients may benefit from a non-invasive detection test like the KIT urine assay as an alternative means of diagnostic workup to ascertain presence of urolithiasis and assess for recurrence.^1^

The novel KIT assay used random forest modeling to identify the relationship between biomarkers for detection of CKD with high sensitivity.^2^ Statistical and machine learning methods trained predictive models for the development of the KIT Score algorithm.^2^ A linear model incorporating the multi-dimensional partition of the assay measurements was developed into the KIT Score.^2^ Of the six biomarkers in the KIT Stone-Score, cell-free (cfDNA) has the greatest impact on the aggregate score, often helping distinguish inflammatory processes and tissue destruction secondary to nephrolithiasis from diabetes, glomerular disease, or hypertension.^2^

The KIT Score could risk-stratify stone recurrence and provide an objective measure of kidney stone burden with a lower limit of detection of 2 mm. ^3^ Higher levels are present in obstructive compared to non-obstructive nephrolithiasis.^3^ Prior studies including 139 urine samples with 54 healthy controls validated the variation of KIT Stone-Score values between healthy non-stone formers and kidney stone patients and identified KIT's ability to detect kidney stone formers.^3^

In this case report, a healthy asymptomatic woman with no prior history of nephrolithiasis was found to have a markedly elevated KIT Stone-Score with a remarkable cfDNA of 465.37 ng/mL. Subsequent workup identified a complete staghorn stone, that with time could have led to loss of kidney function if left untreated. After a PCNL and URS-LL that rendered her stone free, her urinary KIT normalized 6-months post-operatively (Fig. 1B, Table 1).

We compared the peri- and post-procedural abnormal KIT levels from our case report to urine samples from healthy non-stone formers and a patient with history of recurrent kidney stones (Table 1). The patient with current urolithiasis had a significantly higher KIT stone score compared to both healthy non-stone forming subjects and the patient with prior history of urolithiasis (Table 1). In fact, cfDNA, mcfDNA, and total proteins were significantly higher in our patient compared to patients with a prior stone history and healthy non-stone formers. The KIT stone score discriminated between non-stone formers and those with radiologically confirmed stones. The KIT assay may complement currently available tools to predict future recurrence risk and further risk stratify stone formers. In the future, the KIT assay can be adopted as a clinic-based test in settings with limited access to imaging. Furthermore, the KIT score can be integrated into the usual follow-up to help risk stratify stone recurrence and further tailor imaging frequency. Patients and healthcare systems may benefit from decreased need of ultrasound, CT, and imaging technicians.

Currently, there are various biomarkers that have shown potential in detecting urinary calculi. In a prospective study, Castiglione et al. showed that high serum dephosphorylated and uncarboxylated Matrix-Gla-protein (dpucMGP) was associated with less kidney stone formation after adjustment for age, race, and sex.^4^ A prospective longitudinal study assessed urinary galectin 3 C-terminal-S-osteopontin/urinary full-length osteopontin (Gal3C-S-OPN/uFL-OPN) level and urinary full-length osteopontin (uFl-OPN) level in urolithiasis patients during stone treatment and compared the levels between the residual stone group and stone-free group after URS5. 92.8% of the stone-free urolithiasis group had Gal3C-S-OPN/full-length-OPN levels below the cutoff value after URS-LL; 71.4% of the residual-stone urolithiasis group did not show decreased levels after URS-LL.^5^ DpucMGP and urinary OPN, like KIT Stone-Score biomarkers, have shown a clinical potential for stone recurrence monitoring.

## 4 Conclusion

The urinary KIT test detected the presence of a large staghorn stone in an otherwise completely asymptomatic patient. The KIT Stone-Score levels corrected appropriately after surgical intervention. Further larger studies are needed to highlight the utility of the KIT assay as a reliable non‐invasive tool in the detection and monitoring of kidney stone disease.

Consent: Patient informed consent was obtained using the UCSF-IRB approval number (CHR #14–14533).

## Funding

None.

## Declaration of competing interest

M.M.S. is an inventor of the KIT Assay, IP for which is exclusively owned by the Regents, University of California San Francisco and licensed to NephroSant (San Francisco, CA). R.S is a consultant for NephroSant.
